# A giant Brunner gland hamartoma successfully treated by endoscopic excision followed by transanal retrieval: A case report

**DOI:** 10.1097/MD.0000000000025048

**Published:** 2021-04-09

**Authors:** Motonobu Maruo, Tomomitsu Tahara, Fumihiro Inoue, Takeshi Kasai, Natsuko Saito, Kazunori Aoi, Masahiro Takeo, Kimi Sumimoto, Masao Yamashina, Miki Murata, Masanori Koyabu, Takahiro Wakamatsu, Noriyo Yamashiki, Akiyoshi Nishio, Kazuichi Okazaki, Makoto Naganuma

**Affiliations:** a Third Department of Internal Medicine, Kansai Medical University; b Kansai Medical University Kori Hospital, Osaka, Japan.

**Keywords:** anemia, Brunner gland hamartoma, endoscopic resection, transanal retrieval

## Abstract

**Rationale:**

Brunner gland hamartoma (BGH) is a rare tumor of the duodenum. Although BGH is a benign tumor, larger lesion with gastrointestinal symptoms requires tumor removal. We report a giant BGH, successfully treated by endoscopic excision followed by transanal retrieval.

**Patient concerns:**

A 38-year-old woman complained of severe anemia, tarry stool, and vomiting.

**Diagnoses:**

Esophagogastroduodenoscopy (EGD) showed a pedunculated giant submucosal mass at the duodenal bulb.

**Interventions:**

We attempted to remove it because the lesion seemed to be responsible for patient's anemia and vomiting. The lesion had clear but bulky stalk. We carefully cut the stalk using needle-knife and IT knife2. We tried to retrieve specimen, but the mass could not pass through the pyloric ring because of its size. Then we tried to obtain the specimen from anus. Polyethylene glycol solution was administered to accelerate rapid excretion.

**Outcomes:**

The mass was successfully removed and was histologically confirmed as a giant BGH, measuring 55 mm in size.

**Lessons:**

Reports about endoscopic resection of giant BGH are rare. Moreover, our case is the first report of transanal retrieval of resected specimen using polyethylene glycol solution. Endoscopic resection of BGH is less-invasive but can be more challenging if the mass is large. Our case provides useful option for endoscopic treatment of giant BGH.

## 1 Introduction

Brunner gland hamartoma (BGH) is a rare tumor of the duodenum arising from the Brunner glands.^[1]^ Although BGH is a benign tumor, patients who have larger lesion with gastrointestinal symptoms such as bleeding, nausea, vomiting, and abdominal pain,^[2–4]^ require tumor removal. Larger BGHs have been generally treated by surgical intervention,^[5,6]^ while some lesions have been treated by endoscopic resection despite its larger size.^[7,8]^ Achievement of en bloc specimen would be important for histological confirmation of BGH, however, in larger lesions, transoral retrieval of the resected specimen may be difficult because of its size. Here, we report a BGH measuring about 5.5 cm in proximal duodenum, successfully treated by endoscopic excision followed by transanal retrieval.

## 2 Case report

A 38-year-old woman presented with severe anemia, tarry stool, and vomiting. She had no remarkable medical history. Before being referred to our hospital, she visited to a clinic and was diagnosed as severe anemia with 6.3 g/dL hemoglobin by hematological test. She was also informed of a protruding lesion at the duodenal bulb by esophagogastroduodenoscopy (EGD).

On physical examination, pallor was noted, while her abdomen was soft and free from pain and tenderness. Slight fever and tachycardia (temperature 37.2 °C, pulse 117 beats/min) were noted but other vital signs (pressure of 142/99 mmHg, oximetry saturation 100%) were normal. Since oral iron supplement has been offered to the patient for 3 weeks before she was admitted to our hospital, hematological test showed improvement of anemia with 9.7 g/dL hemoglobin. Other laboratory results were within normal limits, including tumor marker levels. *Helicobacter pylori* test was negative.

**Figure 1:**
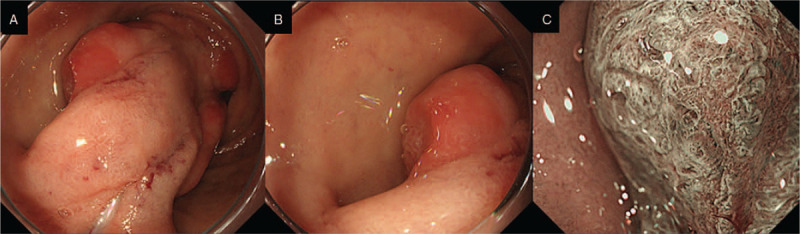
EGD findings. (A) A pedunculated giant submucosal mass seen the anterior wall of the duodenal bulb. At the base of the lesion, a bulky stalk is seen. (B) From the body to the top of the lesion, the mucosal surface revealed congested hyperplastic changes. Erosive appearance was also seen. (C) Magnifying endoscopy with NBI showed dilated villous and pit structures at the erosive area but there was no evidence of endoscopic appearances associated with neoplastic lesions such as irregular surface and capillary patterns. EGD = esophagogastroduodenoscopy, NBI = narrow band imaging.

**Figure 2:**
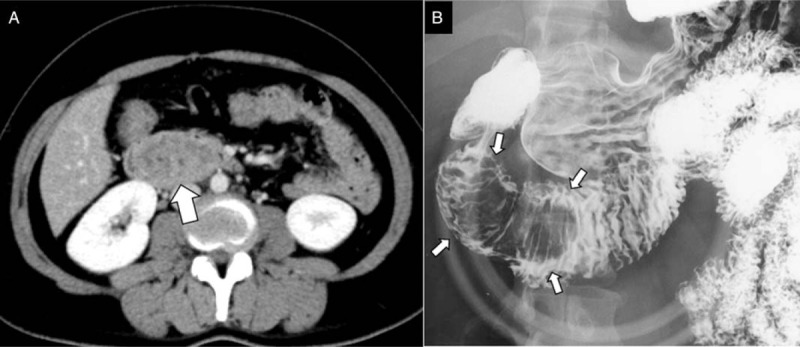
CT analysis (A) and barium x-ray examination (B). A mass measuring about 55 mm in size was shown near the proximal duodenum. (White arrows). CT = computed tomography.

EGD showed a pedunculated giant submucosal mass at the anterior wall of the duodenal bulb (Fig. 1A). From the body to the top of the lesion, congested hyperplastic change and erosive appearance were seen on the mucosal surface (Fig. 1B). Using magnifying endoscopy with narrow band imaging (NBI), dilated villous and pit structures were shown at the erosive area but there was no evidence of neoplastic lesions such as irregular surface and capillary patterns (Fig. 1C). At the base of the lesion, the width of the stalk was >10 mm (Fig. 1A). Computed tomography (CT) analysis and barium x-ray examination also showed a mass near the proximal duodenum measuring about 55 mm in size (Fig. 2A and B). Neither nodal metastasis nor extramural extension was indicated. Although a conclusive diagnosis had not been made for the lesion, we could not rule out the possibility of malignancy. Because the lesion seemed to be responsible for patient's anemia and vomiting, we thought that removing the mass was necessary.

**Figure 3:**
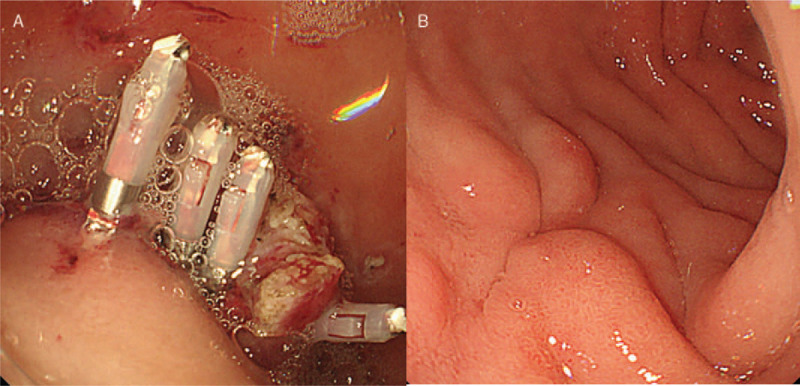
Endoscopic resection. (A) After cutting the stalk of the lesion using needle-knife and IT knife2, hemoclips were immediately placed to prevent bleeding. (B) EGD findings after 2 months showing no evidence of recurrence or tumor remnants. EGD = esophagogastroduodenoscopy.

**Figure 4:**
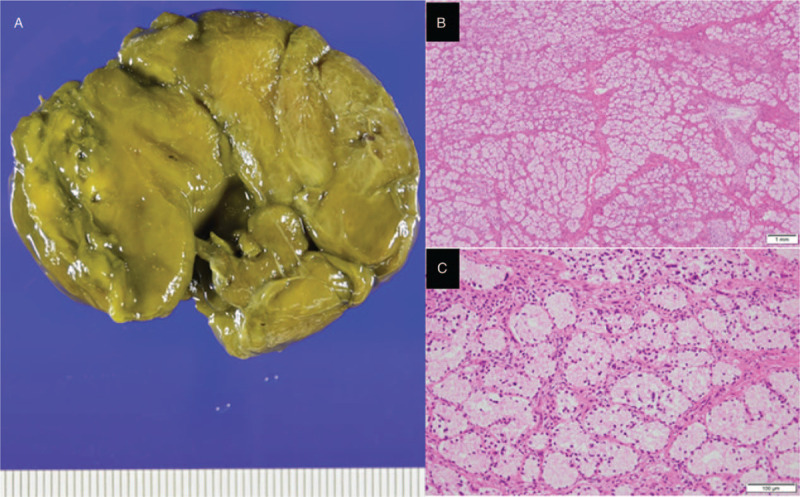
Pathological evaluation of resected specimen. (A) Macroscopic examination showed lobulated mass measuring 5.5 × 4.5 × 2 cm. (B) Histologic section demonstrated mucous cells of the proliferating Brunner glands with basal nuclei without atypia. These proliferating Brunner glands formed lobules of various sizes. The mass was histologically confirmed as a giant BGH. BGH = Brunner gland hamartoma.

Since it was a pedunculated lesion, we attempted to remove it by endoscopic resection. The lesion had clear but bulky stalk. We carefully cut the stalk using needle-knife (Zeon Medical Co., Tokyo, Japan) and IT knife2 (Olympus medical systems Co., Tokyo, Japan). After removing the lesion, hemoclips were immediately placed to prevent bleeding (Fig. 3A). We tried to retrieve the resected specimen from the duodenum to the stomach, but the mass could not pass the pyloric ring because of its size. We finally gave up retrieving it transorally. Instead, we tried to obtain the specimen from the anus. Although the patient had vomiting when she visited to a clinic, there was no evidence of bowel obstruction and the entire lesion was within the duodenum (Fig. 1A). Polyethylene glycol solution (Niflec) was offered to accelerate rapid excretion. The mass was successfully obtained after 10 hours without any complications. The macroscopic examination showed lobulated mass measuring 5.5 × 4.5 × 2 cm (Fig. 4A). The mass was histologically confirmed as a giant BGH (Fig. 4B and C).

No evidence of recurrence or tumor remnants was found by an EDG after 2 months (Fig. 3B). Her hemoglobin rose up to 12.6 g/dL after 3 months. There was also no evidence of recurrence or tumor remnants by an EDG after 12 months (data not shown).

## 3 Discussion

BGH is a benign tumor. However, patients who have larger lesion sometimes present with several symptoms for which we need to consider therapeutic intervention. In our case, the patient presented with severe anemia, tarry stool, and vomiting. In addition, malignancy of the mass could not be excluded by EGD, CT, and barium x-ray examinations. Typical endoscopic appearance of BGH is a protruding submucosal mass with a pedicle,^[1]^ which was also observed in our case. However, other Brunner gland proliferative lesions, such as Brunner gland hyperplasia and adenoma also appear with similar findings. We therefore attempted to remove the lesion for histological evaluation.

BGH can be treated either by endoscopic or surgical excision, for large BGHs, however, surgical resection might be selected because of its ease and safety. Recently, Yi et al^[8]^ reviewed 35 cases of giant BGHs from the literatures and their own case. In these cases, all the exophytic BGH and sessile BGH were removed surgically, while endoscopic resection was performed in the pedunculated BGH. In addition, surgical treatment was also chosen in the pedunculated mass for the following reasons: malignancy of the mass cannot be excluded; some emergency complications like intussusception; the mass was too large, or the stalk was too thick to be removed endoscopically. For the endoscopic resection of pedunculated BGH, endoscopic mucosal resection (EMR) or hot snare polypectomy (HSP) were mainly used.^[7,8]^ However, our case presented bulky stalk, suggesting that EMR and HSP was more challenging. We therefore cut the stalk using needle-knife and IT knife2, which are mainly used for endoscopic submucosal dissection (ESD). Compared with hot snare used for EMR or HSP, ESD devices allow as to carefully adjust the location and speed of mucosal incisions, which can avoid bleeding during the procedure. Some authors reported that pretreating the stalk by placing hemoclips or endoloop would be important to prevent bleeding.^[7,8]^ But we believe that placing these devices limit the appropriate incision line. Therefore, we initially removed the lesion and thereafter placed hemoclips. Although our case was successfully resected without severe bleeding, possibility of massive bleeding should be considered even if we carefully perform submucosal incisions.

Successful retrieval of the resected specimen is also important for histological confirmation of BGH. We initially planned transoral retrieval but it was unsuccessful because the mass was too large to pass the pyloric ring. We therefore decided to obtain the specimen from the anus. We used Polyethylene glycol solution to accelerate rapid excretion. The mass was successfully obtained without any complications and histological evaluation was successful. There have been no cases of BGH lesions transanally retrieved following to endoscopic resection,^[8]^ possibly because endoscopic resection is indicated for relatively smaller BGHs that can be retrieved orally. If the resected specimen could be retrieved from the anal, physician can positively consider endoscopic resection for giant BGH. Our case provides useful information for endoscopists who perform endoscopic resection of giant BGH, but the safety of this procedure needs be further evaluated. It should be noted that the possibility of bowel obstruction cannot be excluded if the mass is larger.

In conclusion, we reported a patient with a giant BGH who was successfully treated by endoscopic excision followed by transanal retrieval. Although BGH is a benign tumor, resection should be considered because large tumors may cause symptoms such as bleeding and pain. Endoscopic resection would be preferable to conventional surgery because of less-invasiveness but would be more challenging if the mass is large. We believe that our case provides salient finding for many endoscopists who plan endoscopic treatment of giant BGH.

## Author contributions

**Conceptualization:** Motonobu Maruo, Tomomitsu Tahara, Fumihiro Inoue, Takeshi Kasai.

**Data curation:** Motonobu Maruo, Tomomitsu Tahara, Fumihiro Inoue, Takeshi Kasai, Natsuko Saito.

**Formal analysis:** Motonobu Maruo.

**Investigation:** Motonobu Maruo, Kazunori Aoi, Masahiro Takeo, Kimi Sumimoto, Masao Yamashina, Miki Murata, Masanori Koyabu, Noriyo Yamashiki.

**Supervision:** Takahiro Wakamatsu, Akiyoshi Nishio, Kazuichi Okazaki, Makoto Naganuma.

**Writing – original draft:** Tomomitsu Tahara.

**Writing – review & editing:** Tomomitsu Tahara.
